# Supplementary material for: Increased lethality in influenza and SARS-CoV-2 coinfection is prevented by influenza immunity but not SARS-CoV-2 immunity
Source: Nat Commun. 2021 Oct 5;12:5819. doi: 10.1038/s41467-021-26113-1 (PMC8492774; doi:10.1038/s41467-021-26113-1)
Supplement: Supplementary file 1 — Supplementary information. [file 41467_2021_26113_MOESM1_ESM.pdf]

Supplementary information for:

Increased lethality in influenza and SARS-CoV-2 coinfection is prevented by influenza immunity but not SARS-CoV-2 immunity

Hagit Achdout[1]*, Einat. B. Vitner[1]*, Boaz Politi[1]*, Sharon Melamed[1]*, Yfat Yahalom-Ronen[1], Hadas Tamir[1], Noam Erez[1], Roy Avraham[1], Shay Weiss[1], Lilach Cherry[1], Erez Bar-Haim[2], Efi Makdasi[1], Didi Gur[2], Moshe Aftalion[2], Theodor Chitlaru[2], Yaron Vagima[2], Nir Paran[1], Tomer Israely[1¶]

Supplementary figure 1:

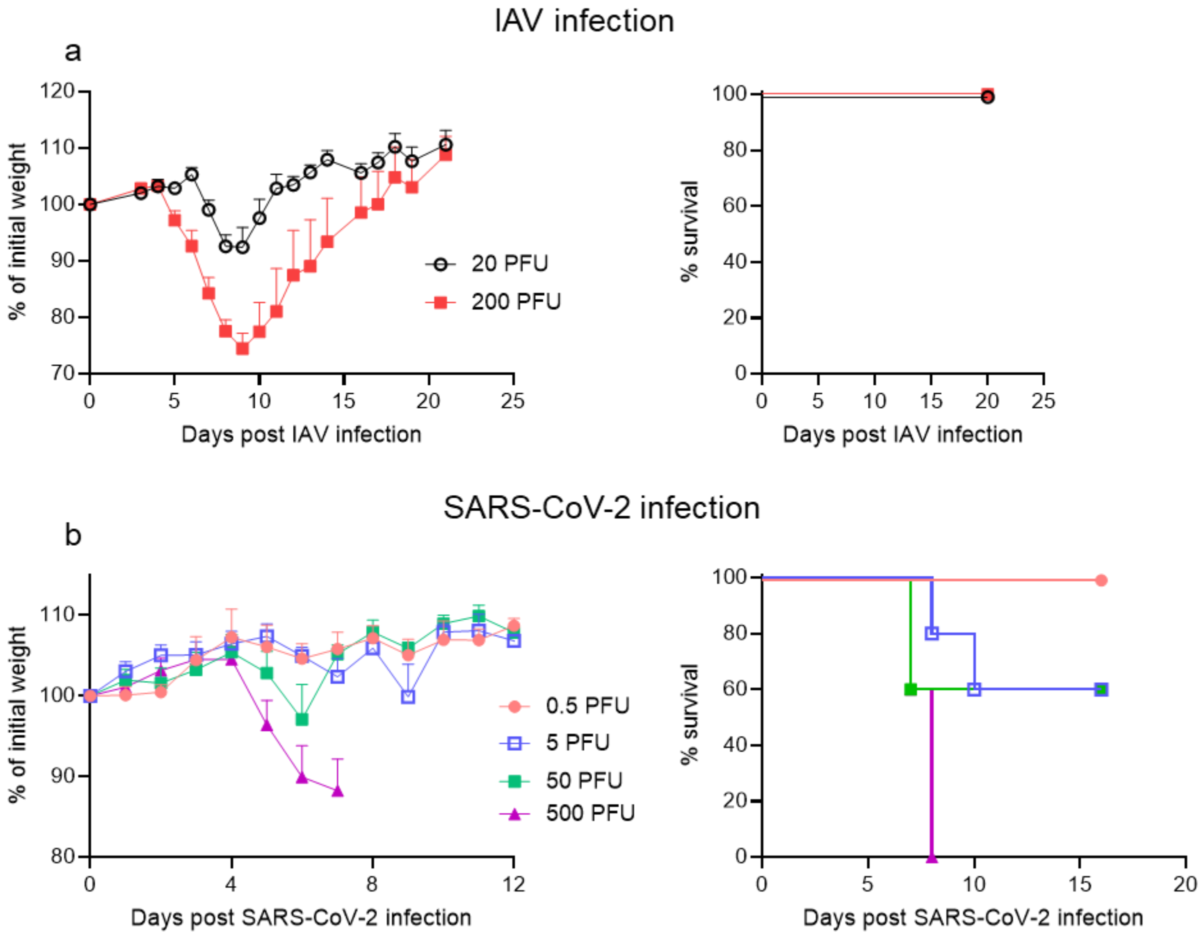

Sup. Fig. 1: K18-hACE2 mice infected with IAV or SARS-CoV-2

(a) K18-hACE2 mice were i.n. infected with 20 or 200 PFU of PR8 virus (n=4 per group).

(b) K18-hACE2 mice were i.n. infected with 0.5-500 PFU of SARS-CoV-2 as indicated. n=4 in the 0.5 PFU group, n=5 in 5-500 PFU groups. Error bars represent the standard error (SE). Source data are provided as a Source Data file.

Supplementary figure 2:

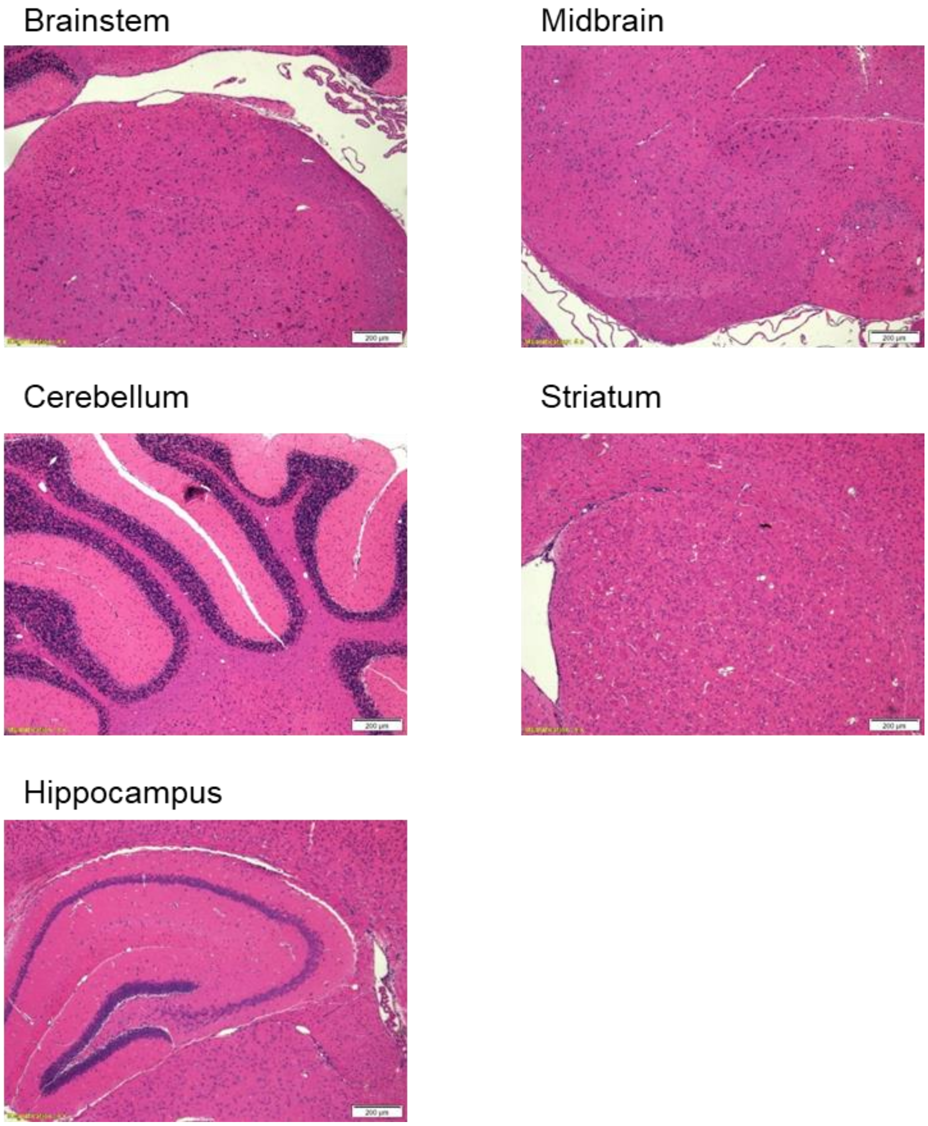

Sup. Fig. 2: Histopathological analysis of IAV and SARS-CoV-2 coinfection in K18-hACE2 mice

H&E staining of brain sections from K18-hACE2 mice coinfected with IAV and SARS-CoV-2 at 6 dpIi (4 dpSi). Scale bar=200 µm. Images are representative of a group of 5 mice.

Supplementary figure 3:

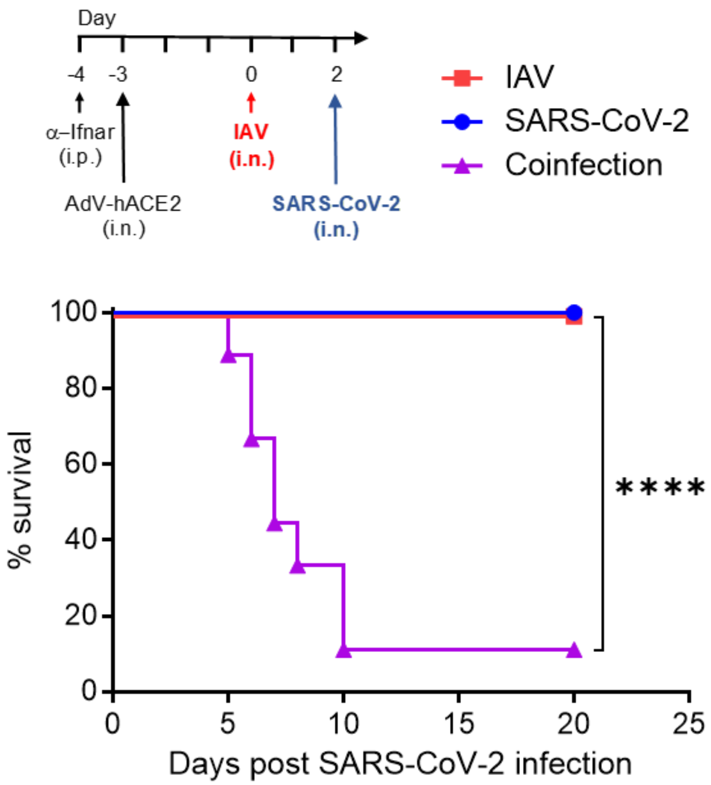

Sup. Fig. 3: Coinfection in AdV-hACE2-transduced mice

Male and female C57BL/6J mice received anti-Ifnar1 mAb injection (2 mg, i.p. route; day -4), AdV-hACE2 instillation (10^8 PFU, i.n. route, day -3), IAV instillation (80 PFU, i.n. route, day 0), and SARS-CoV-2 instillation (10^5 PFU, i.n. route, day +2) as indicated. Survival curve, ****$p<0.0001$, SARS-CoV-2 infection compared to coinfection, ***$p=0.0002$ IAV infection compared to coinfection, log-rank test (Mantel-Cox). The figure includes the IAV-infected group, which consisted of 9 mice; the SARS-CoV-2-infected group, which consisted of 11 mice; and the coinfection group, which consisted of 9 mice. Source data are provided as a Source Data file.

Supplementary figure 4:

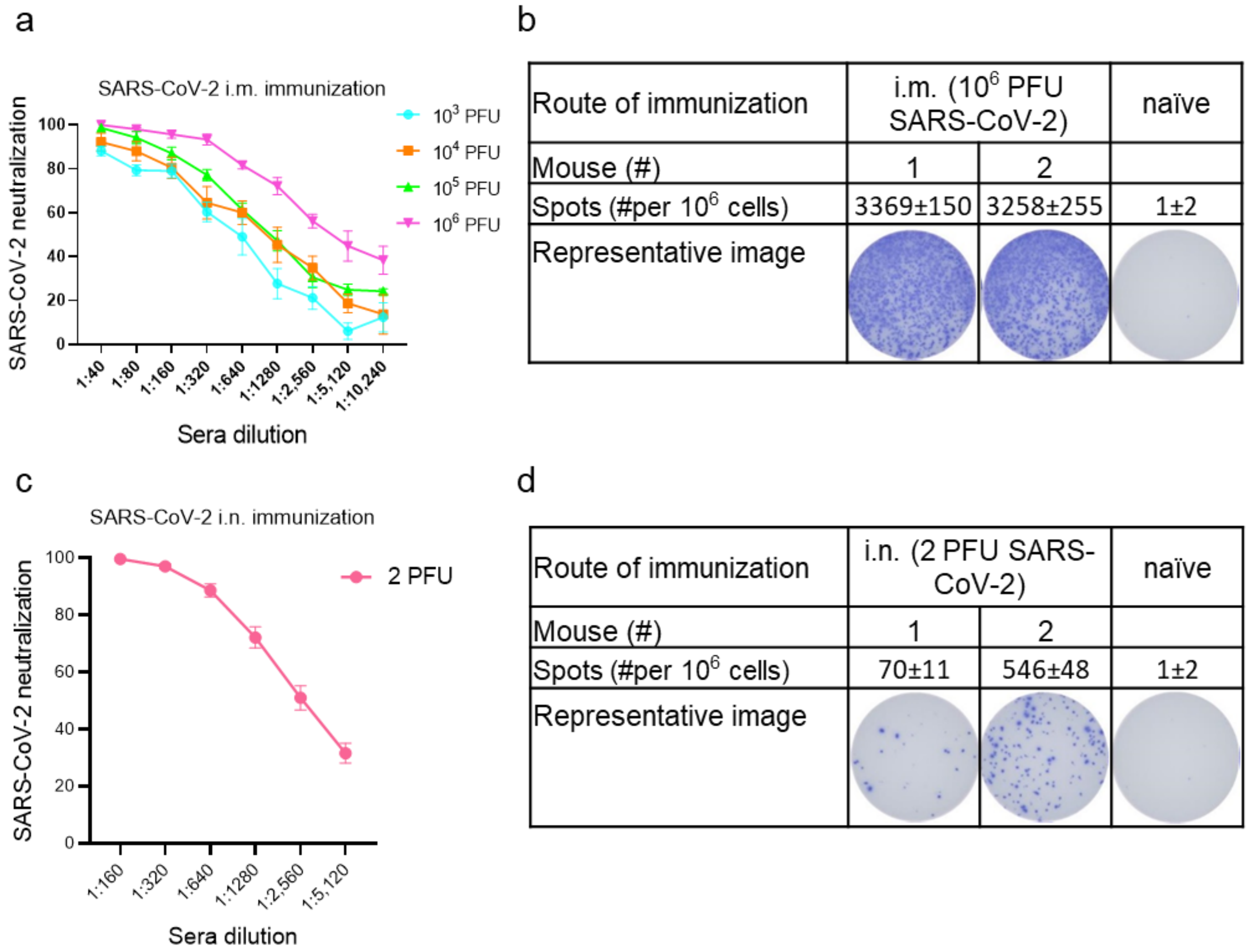

Sup. Fig. 4: SARS-CoV-2 immune response analysis

K18-hACE2 mice were immunized with SARS-CoV-2 by the i.m. route with 10^3-10^6 PFU. (a) Twenty-one days post immunization, serum samples were collected, and the anti-SARS-CoV-2 neutralizing antibody levels were determined. The figure includes 3 mice per dose of SARS-CoV-2. (b) Seven days post immunization, the mice were sacrificed, and ELISpot assays were performed using SARS-CoV-2 peptide. Two distinct mice treated via the i.m. route with 10^6 pfu. For the naïve mice, we used a pool of two naïve mice. Each

sample was measured twice. All measurement data are expressed as the means±SD.

K18-hACE2 mice were immunized with SARS-CoV-2 by the i.n. route with 2 PFU. (c) Twenty-one days post immunization, serum samples were collected, and the anti-SARS-CoV-2 neutralizing antibody levels were determined. The figure includes 20 mice. (d) Seven days post-immunization, the mice were sacrificed, and ELISpot assays were performed using SARS-CoV-2 peptide for two distinct mice treated via the i.n. route with 2 PFU. For the naïve mice, we used a pool of two naïve mice. Each sample was measured twice. All measurement data are expressed as the mean±SD. Source data are provided as a Source Data file.

Supplementary figure 5:

a

Symbol	SARS-CoV-2 immunization	Infection with	n
▽	+	SARS-CoV-2	7
■	-	IAV	6
●	-	SARS-CoV-2	9
▼	+	Coinfection	14
▲	-	Coinfection	9

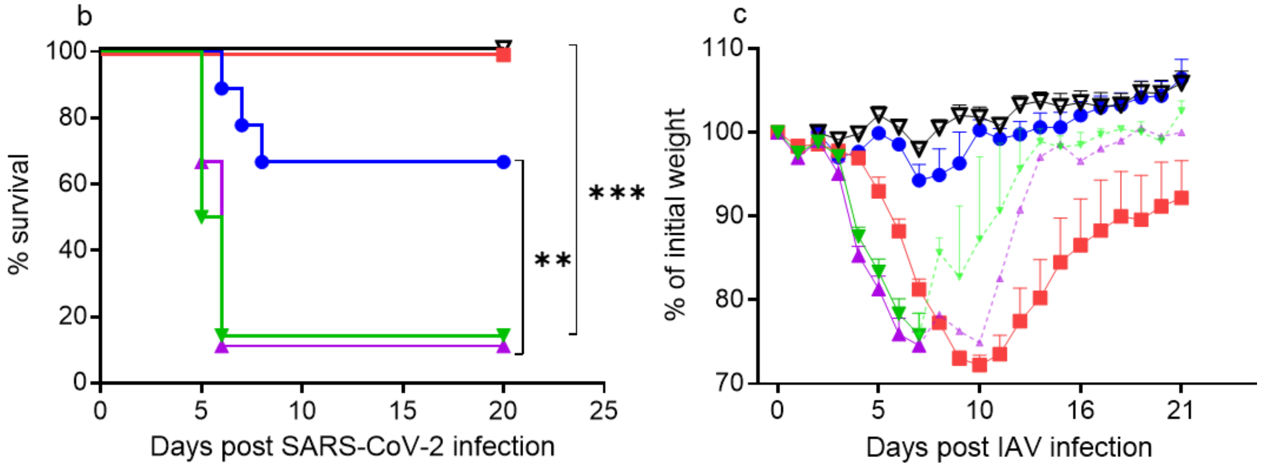

Sup. Fig. 5: SARS-CoV-2 immunization by the i.n. route did not protect coinfected K18-hACE2 mice

(a) Legend for b,c. K18-hACE2 mice were immunized by the i.n. route with 2 PFU SARS-CoV-2. Thirty days post immunization, immunized and nonimmunized mice were infected i.n. with the indicated virus. Survival curve (b) **p=0.0039 (SARS-CoV-2 infection compared to coinfection) ***P=0.0005 (SARS-CoV-2 immunization and infection compared to SARS-CoV-2 immunization and coinfection) log-rank test (Mantel-Cox). Percent weight loss following infection (c). The dashed lines represent 1 or 2 surviving mice of 9 or 14, respectively. Measurement data are expressed as the mean+SE. n, number of mice per group. Source data are provided as a Source Data file.

Supplementary figure 6:

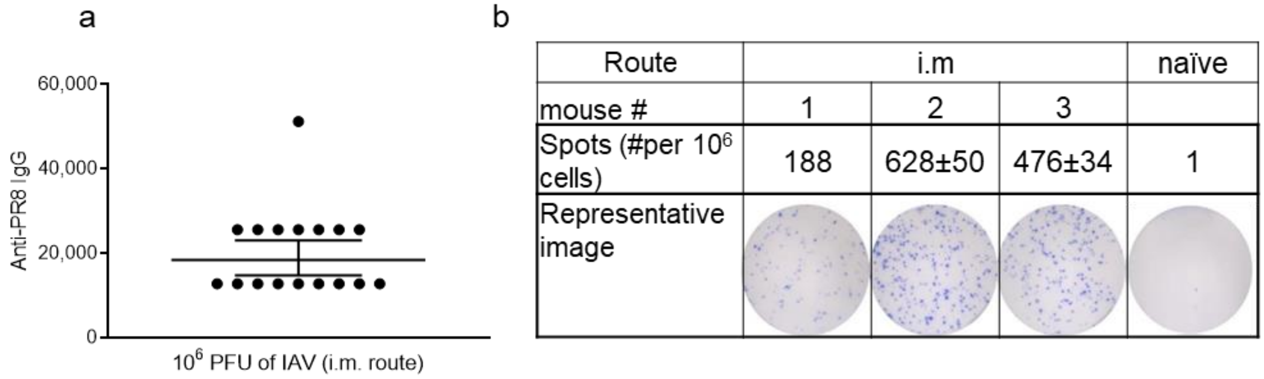

Sup. Fig. 6: IAV immune response analysis

K18-hACE2 mice were immunized with IAV by the i.m. route (10^6 PFU). (a) Twenty-one days post immunization, serum samples were collected, and antibody binding against PR8 virus was determined. The figure includes 17 mice. (b) Twenty-five days post immunization, the mice were sacrificed, and ELISpot assays were performed using PR8 peptides. Three distinct mice receiving IAV immunization were measured. For the naïve mice, we used a pool of two naïve mice. Each sample was measured twice. All measurement data are expressed as the means±SD. Source data are provided as a Source Data file.

Supplementary figure 7:

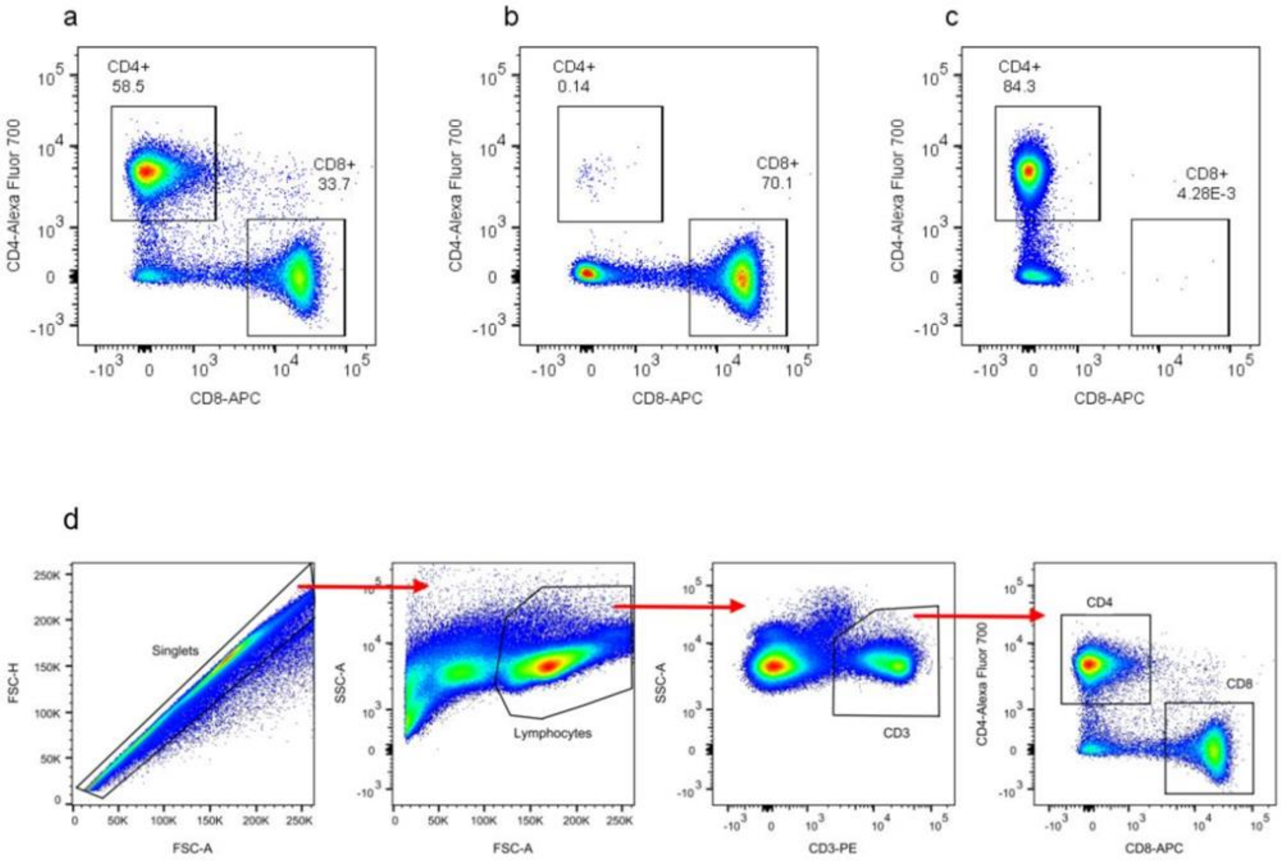

Sup. Fig. 7: Flow cytometry analysis of CD4 and CD8 T cell depletion.

Mice were i.p. administered with PBS (a), 200 μg of anti-CD4 (b) or anti-CD8 (c) mAbs. After twenty-four hours, the mice were sacrificed, and the spleens were harvested for a flow cytometry analysis. Gating strategy for a-c (d).
