# Supplementary material for: Increased lethality in influenza and SARS-CoV-2 coinfection is prevented by influenza immunity but not SARS-CoV-2 immunity
Source: Nat Commun. 2021 Oct 5;12:5819. doi: 10.1038/s41467-021-26113-1 (PMC8492774; doi:10.1038/s41467-021-26113-1)
Supplement: Supplementary file 3 — Reporting summary. [file 41467_2021_26113_MOESM3_ESM.pdf]

nature portfolio

Corresponding author(s): Tomer Israely

Last updated by author(s): Aug 15, 2021

Reporting Summary

Nature Portfolio wishes to improve the reproducibility of the work that we publish. This form provides structure for consistency and transparency in reporting. For further information on Nature Portfolio policies, see our Editorial Policies and the Editorial Policy Checklist.

Statistics

For all statistical analyses, confirm that the following items are present in the figure legend, table legend, main text, or Methods section.

n/a	Confirmed	
☐	☒	The exact sample size (*n*) for each experimental group/condition, given as a discrete number and unit of measurement
☒	☐	A statement on whether measurements were taken from distinct samples or whether the same sample was measured repeatedly
☐	☒	The statistical test(s) used AND whether they are one- or two-sided<br>*Only common tests should be described solely by name; describe more complex techniques in the Methods section.*
☒	☐	A description of all covariates tested
☒	☐	A description of any assumptions or corrections, such as tests of normality and adjustment for multiple comparisons
☐	☒	A full description of the statistical parameters including central tendency (e.g. means) or other basic estimates (e.g. regression coefficient) AND variation (e.g. standard deviation) or associated estimates of uncertainty (e.g. confidence intervals)
☐	☒	For null hypothesis testing, the test statistic (e.g. *F*, *t*, *r*) with confidence intervals, effect sizes, degrees of freedom and *P* value noted<br>*Give P values as exact values whenever suitable.*
☒	☐	For Bayesian analysis, information on the choice of priors and Markov chain Monte Carlo settings
☒	☐	For hierarchical and complex designs, identification of the appropriate level for tests and full reporting of outcomes
☒	☐	Estimates of effect sizes (e.g. Cohen's *d*, Pearson's *r*), indicating how they were calculated

Our web collection on statistics for biologists contains articles on many of the points above.

Software and code

Policy information about availability of computer code

Data collection	N/A
Data analysis	Statistical analysis were performed in GraphPad Prism 8.4.3 and microsoft excel 2016. Figures were prepared using GraphPad 8.4.3

For manuscripts utilizing custom algorithms or software that are central to the research but not yet described in published literature, software must be made available to editors and reviewers. We strongly encourage code deposition in a community repository (e.g. GitHub). See the Nature Portfolio guidelines for submitting code & software for further information.

Data

Policy information about availability of data

All manuscripts must include a data availability statement. This statement should provide the following information, where applicable:

- Accession codes, unique identifiers, or web links for publicly available datasets
- A description of any restrictions on data availability
- For clinical datasets or third party data, please ensure that the statement adheres to our policy

The data that support the finding are available from the corresponding author upon reasonable request

Field-specific reporting

Please select the one below that is the best fit for your research. If you are not sure, read the appropriate sections before making your selection.

☒ Life sciences ☐ Behavioural & social sciences ☐ Ecological, evolutionary & environmental sciences

For a reference copy of the document with all sections, see nature.com/documents/nr-reporting-summary-flat.pdf

Life sciences study design

All studies must disclose on these points even when the disclosure is negative.

Sample size	In this study sample size was selected without pre-calculation. This was done due to the complete lack of previous data concerning the expected diversity in individual animal responses, as well as the group variation in results. We chose groups of n=4-14 for morbidity and mortality. For gene analysis we used groups of 4 mice. For viral load we used groups of 4 or 10 mice. For histopathologicsal analysis we used groups of 5 mice.
Data exclusions	No data was excluded.
Replication	Data shown in Figure 1a,b is representative of 4 repeated experiments.<br>Data shown in Figure 1c,d is representative of 2 repeated experiments.<br>Data shown in Figure 1e,f was performed once.<br>Figure 2a-f were done once on groups of 10 mice.<br>Figure 2g was done once on groups of 5 mice.<br>Figure 3 were done once on groups of 4 mice.<br>Figure 4 were done once on groups of 5-14 mice.<br>Data shown in The Supplementary Figures was performed once.<br>The chosen group size we used allows significant results.
Randomization	All animals were randomly assigned to the experimental groups.
Blinding	In our experience, preliminary animal studies, especially performed under BSL-3 conditions, do not require blinding and thus were not blinded in this case. The identity of each group was essential in the performance of the experiments.

Reporting for specific materials, systems and methods

We require information from authors about some types of materials, experimental systems and methods used in many studies. Here, indicate whether each material, system or method listed is relevant to your study. If you are not sure if a list item applies to your research, read the appropriate section before selecting a response.

Materials & experimental systems

n/a	Involved in the study	
☐	☒	Antibodies
☐	☒	Eukaryotic cell lines
☒	☐	Palaeontology and archaeology
☐	☒	Animals and other organisms
☒	☐	Human research participants
☒	☐	Clinical data
☒	☐	Dual use research of concern

Methods

n/a	Involved in the study	
☒	☐	ChIP-seq
☐	☒	Flow cytometry
☒	☐	MRI-based neuroimaging

Antibodies

Antibodies used	Anti-mouse IgG alkaline phosphatase-conjugated Sigma, Israel, cat. A5153; Anti-mouse CD4, clone GK1.5, ATCC TIB-207; Anti-mouse CD8, clone 2.43, ATCC TIB349 210; PE anti-mouse CD3ε (clone 145-2C11), BD Biosciences, cat. 553063; Alexa Fluor 700 anti-mouse CD4 (clone RM4-5), BD Biosciences, cat. 557956; APC anti-mouse CD8a (clone 56–6.7), BD Biosciences, cat. 553035.
Validation	Secondary antibodies specificity was validated by staining with the secondary antibody without primary antibody, and showing no signal.

Eukaryotic cell lines

Policy information about cell lines

Cell line source(s)	Vero E6 cells (ATCC® CRL-1586TM) and Madin-Darby canine kidney (MDCK) cells (ATCC® CCL-34™)

Authentication	Authentication were performed according to characteristics features as described by ATCC.
Mycoplasma contamination	Cell lines were tested negative for Mycoplasma.
Commonly misidentified lines (See ICLAC register)	No commonly misidentified cell lines were used in the study.

Animals and other organisms

Policy information about studies involving animals; ARRIVE guidelines recommended for reporting animal research

Laboratory animals	K18-hACE2 transgenic mice (B6. Cg234 Tg(K18-ACE2)2Prlmn/J; #034860), Female, 6-8 weeks old. C57BL/6J mice (Jackson Laboratory), female and male, 6-8 weeks old.
Wild animals	The study did not involve wild animals.
Field-collected samples	The study did not involve samples collected in the field.
Ethics oversight	All animal experiments involving SARS-CoV-2 were conducted in a BSL3 facility in accordance with the guideline of the Israel Institute for Biological Research (IIBR) animal experiments committee. Protocol numbers: #M-29-20, M-39-20, M-40-20, M-41-20, M-36-21, M-37-21.

Note that full information on the approval of the study protocol must also be provided in the manuscript.

Flow Cytometry

Plots

Confirm that:

☒ The axis labels state the marker and fluorochrome used (e.g. CD4-FITC).

☒ The axis scales are clearly visible. Include numbers along axes only for bottom left plot of group (a 'group' is an analysis of identical markers).

☒ All plots are contour plots with outliers or pseudocolor plots.

☒ A numerical value for number of cells or percentage (with statistics) is provided.

Methodology

Sample preparation	Spleens from mice were dissociated in GentleMACS C-tubes (Miltenyi Biotec), red blood cells were lysed and splenocytes were stained and analysed by flow cytometry.
Instrument	The samples were collected using a Fortessa flow cytometer (BD Biosciences)
Software	The data was analyzed with FlowJo software version 10 (TreeStar)
Cell population abundance	*Describe the abundance of the relevant cell populations within post-sort fractions, providing details on the purity of the samples and how it was determined.*
Gating strategy	CD4 and CD8 subpopulations were gated out of singlets (FSC-A/FSC-H) lyphocytes (FSC-A/SSC-A), CD3 positive populations.

☒ Tick this box to confirm that a figure exemplifying the gating strategy is provided in the Supplementary Information.
